# Comparison of carotid intima–media thickness and coronary artery calcium score for estimating subclinical atherosclerosis in patients with fatty liver disease

**DOI:** 10.5830/CVJA-2017-052

**Published:** 2018

**Authors:** Kim Hyun-Jin, Park Hyung-Bok, Suh Yongsung, Cho Yoon-Hyeong, Hwang Eui-Seok, Cho Deok-Kyu, Choi Tae-Young

**Affiliations:** Department of Cardiology, Myongji Hospital, Goyang–si, South Korea; Department of Cardiology, Myongji Hospital, Goyang–si, South Korea; Department of Cardiology, Myongji Hospital, Goyang–si, South Korea; Department of Cardiology, Myongji Hospital, Goyang–si, South Korea; Department of Cardiology, Myongji Hospital, Goyang–si, South Korea; Department of Cardiology, Myongji Hospital, Goyang–si, South Korea; Department of Internal Medicine, Cardiovascular Centre, Myongji Hospital, Goyang–si, South Korea

**Keywords:** atherosclerosis, carotid intima–media thickness, coronary artery calcium score, fatty liver

## Abstract

**Introduction:**

Fatty liver disease (FLD) is correlated with cardiovascular disease. Carotid intima–media thickness (CIMT) and coronary artery calcium score (CACS) can noninvasively identify subclinical atherosclerosis and predict risk for cardiovascular events. This study evaluated CIMT and CACS measurements to detect subclinical atherosclerosis in patients with and without FLD.

**Methods:**

Patients who underwent carotid and abdominal ultrasounds as well as cardiac computed tomography (CT) scans were evaluated retrospectively. The differences between the mean CIMT value and CACS measurements in patients with FLD and those with normal livers were estimated.

**Results:**

Among 819 patients (average age of 53.3 ± 11.2 years), 330 had FLD. The CIMT was greater in patients with FLD compared to the controls (0.79 ± 0.17 vs 0.76 ± 0.17 mm, p = 0.012), and carotid plaques were more commonly seen in patients with FLD. The incidence of a composite of larger CIMT (≥ 75th percentile) plus plaque presence was higher in FLD patients (43.3 vs 36.0%, p = 0.041). Particularly among young patients (≤ 50), the CIMT was larger in patients with FLD than in the controls. FLD increased the risk of a composite of large CIMT plus plaque presence in young patients (odds ratio 1.92, 95% confidence interval 1.05–3.49, p = 0.034). However, patients with FLD had no greater incidence of CACS of over 100 than the controls.

**Conclusion:**

CIMT was a better marker of underlying subclinical atherosclerotic risk among patients with FLD than CACS. FLD particularly, increases the risk of subclinical atherosclerosis in patients younger than 50 years of age. These patients should undergo screening CIMT to detect atherosclerosis and modify risk factors.

Fatty liver disease, a common hepatic manifestation of the metabolic syndrome, is linked to an increased risk for cardiovascular disease and is proposed to be an independent risk factor for cardiovascular disease.^1^–^3^ Patients with fatty liver disease also have increased cardiovascular mortality rates regardless of other traditional risk factors,^4^ and have increased incidence of subclinical atherosclerosis.^3^ Although the biological mechanism that explains the relationship between fatty liver disease and atherosclerosis has not been fully proven, recent studies have shown that it may be related to hepatic insulin resistance, chronic inflammation, oxidative stress and dyslipidaemia, including low adiponectin levels.^5^–^7^

Carotid intima–media thickness (CIMT), as measured by carotid ultrasound, has been used as a surrogate measurement of subclinical atherosclerosis.^8^ This measurement is correlated with risk for cardiovascular events.^9^ Coronary artery calcium score (CACS), as measured by cardiac computed tomography (CT) scan, is also a known marker of atherosclerosis, and the clinical risk for all–cause mortality and cardiovascular disease events increases with increasing CACS.10 In addition, CACS over 100 is a known predictor of coronary events.^11^,^12^

Although previous studies have shown that fatty liver disease is associated with coronary artery calcification,^13^,^14^ there are no specific guidelines recommending screening for subclinical atherosclerosis in patients with fatty liver disease. Further evaluations should assess the progression of atherosclerosis in young patients with fatty liver disease, even in the absence of other traditional risk factors.

This study evaluated the efficacy of CIMT measurements and CACS in detecting subclinical atherosclerosis in patients with fatty liver disease.

## Methods

This was a retrospective cohort study and the sample was made up of patients who visited our healthcare centre to undergo routine healthcare maintenance evaluations between June 2011 and December 2013 (n = 23 474). Inclusion criteria were performance on the same day of carotid and abdominal ultrasounds as well as cardiac CT scans evaluating for coronary calcifications (n = 1 064). Patients were excluded from the study if they had conditions that could lead to chronic liver disease, including hepatitis B surface antigen positivity (n = 60), hepatitis C antibody positivity (n = 6), or excessive alcohol consumption (≥ 20 g/day)15 (n = 179).

The study population was composed of 819 patients. Their clinical features and laboratory findings were collected using electronic medical records.

The study was approved by the local institutional review board and was conducted according to the Declaration of Helsinki. The institutional review board exempted written informed patient consent (MJH 2015–01–068).

Carotid artery examination was performed using a Vivid E9 ultrasound system (GE Healthcare, Little Chalfont, UK) and an 11L linear probe. Mean CIMT measurements were performed by an experienced ultrasonographer on the far wall of both common carotid arteries at end–diastole along an arterial segment of 10 mm in length located 10 mm proximal to the carotid bulb, using semi–automated border detection software. Carotid plaques were defined as focal and isolated areas of abnormal intima protruding into the lumen, greater than 15 mm or 50% of the surrounding IMT value.^16^ Carotid plaque–free segments were evaluated for CIMT analysis.

The mean CIMT value was calculated by averaging the CIMT measurements of the left and right common carotid arteries. For evaluating carotid plaque, the common carotid arteries, carotid bifurcations and external and internal carotid arteries were scanned. We also evaluated the incidence of a composite of a CIMT value higher than the 75th percentile plus the presence of carotid plaque. We defined this composite as subclinical atherosclerosis. The 75th percentile values of the mean CIMT value were estimated according to gender.

Abdominal ultrasound is the most commonly used imaging tool for diagnosing fatty liver disease.^17^ Abdominal ultrasound examination was performed by an experienced ultrasonographer using an Acuson Sequoia 512 ultrasound system (Siemens Medical Solutions, USA) and a 4C1 curved probe. Normal liver echogenicity was equal to the echogenicity of the cortex of the right kidney.^18^ Fatty liver disease was diagnosed if the liver echogenicity was diffusely increased compared to the cortex echogenicity of the right kidney.^19^,^20^

Calcium score CT was performed to evaluate for coronary artery calcifications (GE LightSpeed VCT, USA). CT images were obtained with a 2.5–mm slice thickness from the carina to the bottom of the heart. The CACS from all calcified plaques in the coronary tree was calculated by an automated program according to the Agatston method.^21^ We also evaluated the incidence of a CACS over 100, which was a threshold in a previous study, known to increase the risk of atherosclerotic cardiovascular disease.^22^

## Statistical analysis

All data were summarised as frequencies and percentages or means and standard deviations. The laboratory findings of liver function and lipid profiles were summarised as median and interquartile range. The Pearson chi–square test was used to compare categorical variables. The Student’s t–test was used to compare continuous variables and the Mann–Whitney U–test was used when the sample size of at least one group was less than 30. The mean CIMT value, CACS value and the presence of carotid plaques were stratified by age.

Univariate followed by multivariate logistic regression analyses were performed to evaluate the association between subclinical atherosclerosis and fatty liver disease, with adjustments for individuals following traditional risk factors for atherosclerosis: age, hypertension, diabetes and dyslipidaemia. A p–value of less than 0.05 was considered statistically significant. All analyses were performed using SPSS 18.0 (SPSS Inc, Chicago, IL).

## Results

Among a total of 819 patients (mean age: 53.3 ± 11.2 years old) who met the inclusion criteria for this study, 330 (40.3%) patients had fatty liver disease. Patients’ baseline characteristics are presented in Table 1. Patients with fatty liver disease had significantly larger waist and hip circumferences and body mass indices than patients without fatty liver disease. In addition, patients with fatty liver disease had a higher incidence of medical co–morbidities, including hypertension, diabetes and dyslipidaemia and had worse clinical laboratory findings, including haemoglobin A1c, homocysteine, total cholesterol, triglycerides, low–density lipoprotein cholesterol, aspartate aminotransferase, alanine aminotransferase, gamma–glutamyl transpeptidase and alkaline phosphatase levels than patients without fatty liver disease. 

**Table 1:** Baseline patient characteristics

	*All (n = 819)*	*Fatty liver disease (n = 330)*	*Normal livers (n = 489)*	*p-value*
Age (years)	53.25 ± 11.20	53.44 ± 10.87	53.13 ± 11.42	0.698
Male, n (%)	415 (50.7)	212 (64.2)	206 (41.5)	< 0.001
Waist circumference (cm)	81.66 ± 9.39	87.07 ± 7.88	77.99 ± 8.53	< 0.001
Hip circumference (cm)	94.56 ± 6.14	97.02 ± 6.02	92.89 ± 5.65	< 0.001
Waist-to-hip ratio	0.86 ± 0.07	0.90 ± 0.06	0.84 ± 0.07	< 0.001
BMI (kg/m2)	25.07 ± 3.41	26.88 ± 3.19	23.85 ± 2.98	< 0.001
SBP (mmHg)	121.95 ± 13.11	125.9 ± 12.38	119.25 ± 12.91	< 0.001
DBP (mmHg)	74.67 ± 9.80	77.95 ± 9.12	72.46 ± 9.63	< 0.001
Previous history				
Hypertension, n (%)	263 (32.1)	141 (42.17)	122 (24.9)	< 0.001
Diabetes, n (%)	108 (13.2)	70 (21.2)	38 (7.8)	< 0.001
Dyslipidaemia, n (%)	263 (32.1)	141 (42.7)	122 (24.9)	< 0.001
Fasting blood glucose	99.54 ± 18.95	105.04 ± 20.85	95.82 ± 16.57	< 0.001
(mg/dl)	(5.52 ± 1.05)	(5.83 ± 1.16)	(5.32 ± 0.92)	
HbA_1c _(%)	5.75 ± 0.68	5.96 ± 0.78	5.62 ± 0.57	< 0.001
Homocysteine (μmol/l)	10.5 (8.9–12.4)	11.1 (9.3–13.2)	10.2 (8.7–12.0)	< 0.001
Total cholesterol (mg/dl)	191.0 (170.0–214.0)	194.0 (174.0–218.0)	186.0 (167.0–211.5)	0.001
(mmol/l)	[4.95 (4.40–5.54)]	[5.02 (4.51–5.65)]	[4.82 (4.33–5.48)]	
Triglycerides (mg/dl)	115.0 (77.0–17.01)	146.0 (102.8–212.8)	95.0 (59.0–143.0)	< 0.001
(mmol/l)	[1.30 (0.87–0.19)]	[1.65 (1.16–2.40)]	[1.07 (0.67–1.62)]	
LDL cholesterol (mg/dl)	112.0 (93.0–131.0)	116.5 (98.0–134.0)	107.0 (91.0–129.0)	0.001
(mmol/l)	[2.90 (2.41–3.39)]	[3.02 (2.54–3.47)]	[2.77 (2.36–3.34)]	
AST (IU/l)	23.0 (19.0–27.0)	25.0 (20.0–31.0)	21.0 (18.0–26.0)	< 0.001
ALT (IU/l)	20.0 (14.0–29.0)	27.0 (19.0–40.3)	17.0 (13.0–23.0)	< 0.001
Gamma-GTP (IU/l)	25.5 (17.0–42.0)	35.5 (24.0–59.0)	21.0 (15.0–31.0)	< 0.001
ALP (IU/l)	83.0 (53.0–193.0)	85.0 (56.8–201.3)	79.0 (52.0–182.0)	0.023

BMI: body mass index; SBP: systolic blood pressure; DBP: diastolic blood pressure; HbA_1c_: haemoglobin A1c; LDL cholesterol: low-density lipoprotein cholesterol; AST: aspartate aminotransferase; ALT: alanine aminotransferase; Gamma-GTP: gamma-glutamyl transpeptidase; ALP: alkaline phosphatase.

Of the 819 patients, the mean CIMT was 0.77 ± 0.17 mm; 194 (23.7%) patients had carotid plaques (Table 2). The CIMT was significantly higher in patients with fatty liver disease than among patients with normal livers (0.79 ± 0.17 vs 0.76 ± 0.17 mm, p = 0.012). Carotid plaques were identified more commonly in patients with fatty liver disease, but did not reach statistical significance (27.0 vs 21.7%, p = 0.094). The incidence of a composite of larger CIMT (≥ 75th percentile) plus the presence of carotid plaque was significantly higher in patients with fatty liver disease (43.3 vs 36.0%, p = 0.041). The 75th percentile CIMT value of male patients was 0.92 mm and that of female patients was 0.88 mm.

**Table 2:** Difference in CIMT and CACS between the two groups

	All (n = 819)	Fatty liver disease (n = 330)	Normal liver (n = 489)	p-value
CIMT (mm)	0.77 ± 0.17	0.79 ± 0.17	0.76 ± 0.17	0.012
Presence of plaque, n (%)	195 (23.8)	89 (27.0)	106 (21.7)	0.094
CIMT ≥ 75th percentile or presence of plaque, n (%)	319 (38.9)	143 (43.3)	176 (36.0)	0.041
Cardiac CT calcium score	53.07 ± 250.14	73.85 ± 323.29	39.05 ± 184.20	0.077
Cardiac CT calcium score > 100, n (%)	73 (8.9)	32 (9.7)	41 (8.4)	0.518

CIMT: carotid intima–media thickness; CACS: coronary artery calcium score.

Among 819 patients, 561 (68.5%) had a CACS of zero. The mean CACS was 53.07 ± 250.14 (Table 2). Conversely, there were no significant differences in the mean CACS and in the incidence of a CACS greater than 100 between patients with fatty liver disease and those with normal livers.

Table 3 shows the mean CIMT values, the presence of carotid plaques, and the CACS according to the age groups. Interestingly, among patients under 50 years old (n = 310), the CIMT value was significantly higher in the group with fatty livers than among those with normal livers. These young patients with fatty liver disease had increased risk of subclinical atherosclerosis [odds ratios (OR) 1.92, 95% confidence interval (CI): 1.05–3.49, p = 0.034]. After adjustment for age, hypertension, diabetes and dyslipidaemia, fatty liver disease also increased the risk of subclinical atherosclerosis in young patients (OR 1.90, 95% CI: 1.01–3.59, p = 0.047].

However, there were no significant differences in CACS and carotid plaque presence among patients with fatty liver disease compared to those with normal livers according to age group. Young patients with fatty liver disease did not have a significantly increased incidence of CACS > 100 (OR 0.79, 95% CI: 0.14–4.37, p = 0.785) or incidence of carotid plaque presence (OR 1.65, 95% CI: 0.74–3.70, p = 0.221).

**Table 3:** CIMT, carotid plaque and CACS according to age group

*Age group, years (n)*	*Fatty liver disease*	*Normal liver*	*p-value*
CIMT (mm) (n)
< 30 (20)	0.66 ± 0.11 (4)	0.54 ± 0.11 (16)	0.056
31–40 (98)	0.70 ± 0.15 (43)	0.63 ± 0.14 (55)	0.022
41–50 (192)	0.76 ± 0.17 (73)	0.69 ± 0.16 (119)	0.006
51–60 (295)	0.80 ± 0.16 (123)	0.80 ± 0.15 (172)	0.951
61–70 (158)	0.85 ± 0.15 (65)	0.85 ± 0.14 (93)	0.936
> 70 (56)	0.89 ± 0.14 (22)	0.87 ± 0.16 (34)	0.661
CACS			
< 30	0	0	–
31–40	1.21 ± 4.06	2.25 ± 14.62	0.650
41–50	16.62 ± 73.57	18.37 ± 133.82	0.918
51–60		82.89 ±412.46	28.67 ± 117.79	0.158
61–70	93.32 ± 300.54	94.85 ± 337.18	0.977
> 70	311.00 ± 521.03	89.18 ± 197.92	0.028
Carotid plaque, n (%)
< 30	0	0	–
31–40	3 (7.0)	4 (7.3)	1.000
41–50	10 (13.7)	9 (7.6)	0.167
51–60	32 (26.0)	36 (20.9)	0.306
61–70	32 (49.2)	40 (43.0)	0.440
> 70	12 (54.4)	17 (50.0)	0.740

**Fig 1.**
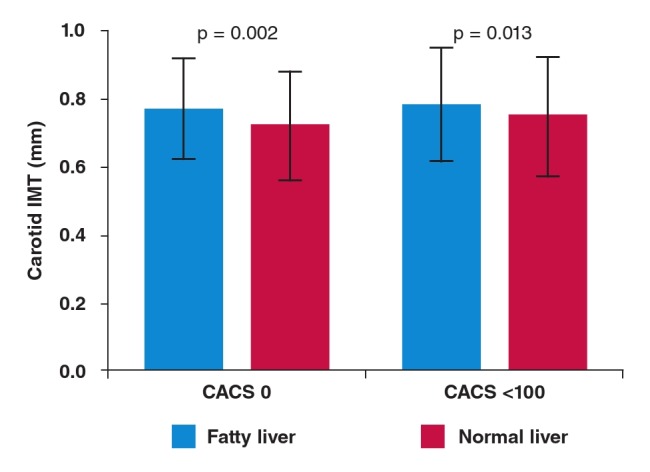
CIMT values according to the presence of fatty liver disease in patients with a CACS of zero and less than 100. The mean CIMT value was significantly higher among patients with fatty liver disease compared to those with normal livers in both groups. CACS: coronary artery calcium score; CIMT: carotid intima–media thickness.

Of the patients with a CACS of zero (n = 561), the patients with fatty liver disease (n = 212) had a significantly higher mean CIMT value than the patients with normal livers (n = 349) (0.77 ± 0.15 vs 0.72 ± 0.16 mm, p = 0.002) (Fig. 1). In addition, among patients with a CACS under 100, the mean CIMT value was also significantly higher among patients with fatty liver disease (n = 298) compared to those with normal livers (n = 448) (0.78 ± 0.17 vs 0.75 ± 0.17 mm, p = 0.013).

## Discussion

The clinical characteristics of patients with fatty liver disease were worse than those of patients with normal livers in our study. The carotid ultrasound images reflected these findings that an increased mean CIMT value was associated with fatty liver disease, and that a composite incidence of larger CIMT (≥ 75th percentile) plus the presence of carotid plaque was also associated with fatty liver disease. Interestingly, young patients (less than 50 years of age) with fatty liver disease showed an increased risk of subclinical atherosclerosis proven by carotid ultrasound rather than by CACS. CIMT was a sensitive marker in identifying atherosclerosis in patients with fatty liver disease, even with a CACS of zero or less than 100.

The pathogenesis of fatty liver disease has been not fully elucidated, but insulin resistance and subclinical inflammation are known to be key mechanisms in the development of fatty liver disease.^3^ Fatty liver disease and the metabolic syndrome share many pathophysiological mechanisms and co–morbidities, such as dyslipidaemia, type 2 diabetes mellitus, insulin resistance and obesity.

As demonstrated in our study, patients with fatty liver disease had more metabolic co–morbidities than those without fatty liver disease. The metabolic syndrome promotes the progression of atherosclerosis and increases the risk of cardiovascular disease.^23^ Moreover, fatty liver disease has been found to be associated with increased mortality rates due to cardiovascular disease and was an independent risk factor for atherosclerosis.^24^,^25^ The association of fatty liver disease with the development of cardiovascular disease indicates the importance of early detection and close follow up of atherosclerosis in patients with fatty liver disease.

The goal of clinical medicine is to prevent as well as cure disease. However, guidelines do not exist regarding which method of screening should be performed in patients with fatty liver disease and how often they should be evaluated to prevent complications caused by atherosclerosis. Prior studies have shown that the measurement of CIMT using carotid ultrasound and of CACS using cardiac CT can detect subclinical atherosclerosis in fatty liver disease patients.^9^,^26^

Increased CIMT in the carotid artery reflects the onset of early atherosclerotic change in the arterial wall. It is known that CIMT measurement by carotid ultrasound in asymptomatic individuals can independently predict future cardiovascular events.^27^,^28^ Importantly, by showing a significant increase in CIMT values in patients with fatty liver disease compared to those with a normal liver, our study demonstrated that the development of subclinical atherosclerosis had already been initiated in patients under 50 years of age with fatty liver disease. In addition, it revealed that CIMT evaluation can effectively detect subclinical atherosclerosis in patients with a CACS of zero or below 100. These findings have important implications for screening and prevention of cardiovascular disease in asymptomatic young patients.

An elevated CACS is also an independent risk factor for coronary artery disease.^22^ Moreover, as coronary artery calcification is associated with a higher incidence of major and minor cardiovascular events, CACS estimation may serve as an important tool in cardiovascular risk assessment. Because arterial calcification represents end–stage changes in vascular atherosclerosis,^29^ the absence of calcifications does not mean that the artery is free of atherosclerosis or non–calcified plaque. Our study also suggests that there was no significant difference in the CACS or in the presence of carotid plaques between patients with fatty liver disease and those with normal livers, despite a difference in CIMT values. Prior studies have also demonstrated that coronary artery calcification was more strongly correlated with carotid plaque burden than with CIMT values in patients with asymptomatic subclinical atherosclerosis.^30^,^31^

In earlier studies, the CACS has been shown to be the best predictor of total cardiovascular disease, while the CIMT or presence of carotid plaque have been found to be slightly better than the CACS in predicting cerebrovascular events.^32^–^34^ Both cardiovascular and cerebrovascular events can be especially catastrophic for young patients with underlying metabolic disease. Therefore, a sensitive method for early detection of subclinical atherosclerosis is needed for patients with fatty liver disease in order to predict the likelihood of vascular complications and to intervene with preventative therapies.

The main limitation of this study is that inclusion required that patients had all examinations performed, including carotid and abdominal ultrasound and calcium score CT, therefore our results may not be generalisable to other subjects with the same clinical characteristics. Another limitation of this study is its crosssectional design. A long–term, causal study is needed to assess the impact of atherosclerosis screening on patient outcomes.

## Conclusion

CIMT was a better marker of underlying subclinical atherosclerotic risk among patients with fatty liver disease than CACS. The measurement of CIMT was especially useful in evaluating the risk of subclinical atherosclerosis in young patients less than 50 years of age. Young patients with fatty liver disease should undergo screening CIMT to detect atherosclerosis so that their risk factors can be modified.
